# Factors associated with smoking cessation in patients with coronary heart disease: a cohort analysis of the German subset of EuroAspire IV survey

**DOI:** 10.1186/s12872-020-01429-w

**Published:** 2020-03-30

**Authors:** D. Goettler, M. Wagner, H. Faller, K. Kotseva, D. Wood, R. Leyh, G. Ertl, W. Karmann, P. U. Heuschmann, S. Störk, on behalf of the German EUROASPIRE IV collaborators, Kim Nolte, Martin Schich, Valerie Wahl, Margret Breunig, Kerstin Eichstädt, Andre Gerhardt, Timo Ludwig, Yvonne Memmel, Anika Quilitzsch, Dominik Schmitt, Theresa Tiffe

**Affiliations:** 1 Comprehensive Heart Failure Center, University and University Hospital Würzburg, Am Schwarzenberg 15, Haus A15, D-97078 Würzburg, Germany; 2 Institute for Clinical Epidemiology and Biometry, University of Würzburg, Würzburg, Germany; 3 Department of Pediatrics, University Hospital of Würzburg, Würzburg, Germany; 4 National Institute for Prevention and Cardiovascular Health, National University of Ireland, Galway, Ireland; 5 Imperial College Healthcare NHS Trust, London, UK; 6 Department of Thoracic and Cardiovascular Surgery, University Hospital Würzburg, Würzburg, Germany; 7 Department of Internal Medicine I, University and University Hospital of Würzburg, Würzburg, Germany; 8 Department of Medicine, Klinik Kitzinger Land, Kitzingen, Germany; 9 Clinical Trial Center, University Hospital of Würzburg, Würzburg, Germany

**Keywords:** Tobacco smoking, Smoking cessation, Coronary heart disease, Secondary prevention, Cardiac rehabilitation

## Abstract

**Background:**

Tobacco smoking is one of the most important risk factors of coronary heart disease (CHD). Hence, smoking cessation is considered pivotal in the prevention of CHD. The current study aimed to evaluate smoking cessation patterns and determine factors associated with smoking cessation in patients with established CHD.

**Methods:**

The fourth European Survey of Cardiovascular Disease Prevention and Diabetes investigated quality of CHD care in 24 countries across Europe in 2012/13. In the German subset, smoking cessation patterns and clinical characteristics were repetitively assessed a) during index event due to CHD by medical record abstraction, b) as part of a face-to-face interview 6 to 36 months after the index event (i.e. baseline visit), and c) by telephone-based follow-up interview two years after the baseline visit. Logistic regression analysis was performed to search for factors determining smoking status at the time of the telephone interview.

**Results:**

Out of 469 participants available for follow-up, 104 (22.2%) had been classified as current smokers at the index event. Of those, 65 patients (62.5%) had quit smoking at the time of the telephone interview, i.e., after a median observation period of 3.5 years (quartiles 3.0, 4.1). Depressed mood at baseline visit and higher education level were less prevalent amongst quitters vs non-quitters (17.2% vs 35.9%, *p* = 0.03 and 15.4% vs 33.3%, p = 0.03), cardiac rehabilitation programs were more frequently attended by quitters (83.1% vs 48.7%, *p* < 0.001), and there was a trend for a higher prevalence of diabetes at baseline visit in quitters (37.5% vs 20.5%, *p* = 0.07). In the final multivariable model, cardiac rehabilitation was associated with smoking cessation (OR 5.19; 95%CI 1.87 to 14.46; *p* = 0.002).

**Discussion:**

Attending a cardiac rehabilitation program after a cardiovascular event was associated with smoking cessation supporting its use as a platform for smoking cessation counseling and relapse prevention.

## Background

Smoking remains a leading risk factor for a variety of diseases and belongs to the most important causes of preventable death [1]. Coronary heart disease (CHD) was the leading single cause of death in the year 2015 [2]. Thirteen smokers need to quit to save one life by smoking cessation after myocardial infarction [3]. The risk of recurrent myocardial infarction is markedly decreased by smoking cessation compared to persistent smoking [4]. Hence, smoking cessation is a pivotal, guideline-supported recommendation in the setting of both primary and secondary prevention of CHD [5]. After an acute cardiac event, approximately half of prior smokers quit smoking [6]. Successful cessation is predominantly driven by discharge recommendations for cardiac rehabilitation, absence of depressed mood and coronary surgery during index hospitalization [7, 8].

For the entire dataset of the fourth European Action on Secondary and Primary Prevention by Intervention to Reduce Events (EuroAspire) IV [9], rates of current smoking at the CHD index event were reported for 16.0%, and persistent smoking 6 to 36 months later for 48.6%. The present study aimed to expand this observation period for the German subset enrolled in EuroAspire IV by another two years in order to study the sustainability of cessation patterns and identify factors associated with smoking cessation.

## Methods

### Study population and design

The fourth European Action on Secondary and Primary Prevention by Intervention to Reduce Events “EuroAspire IV” was a cross-sectional survey conducted at 78 centers in 24 European countries between May 2012 and April 2013 by the European Society of Cardiology. Detailed information on the EuroAspire IV study methodology has been provided previously [9]. Briefly, inclusion criteria, applicable to the index event, were: first or recurrent clinical diagnosis of elective or emergency coronary artery bypass graft (CABG), elective or emergency percutaneous coronary intervention (PCI), acute myocardial infarction (AMI) or acute myocardial ischemia without infarction (AMIsch; troponin negative), and age 18 to 79 years at the date of index event. For the German subset of EuroAspire IV, patients admitted to the University Hospital of Würzburg or the hospital Klinik Kitzinger Land were included. Eligible subjects were identified using appropriate search algorithms applied to the hospital information system and then invited to and examined at the study sites within 6 to 36 months after the CHD index event [10]. All subjects provided written informed consent prior to any study-related investigation. Trained research staff retrospectively extracted all relevant information applying to the index event of study participants from medical records. Further, study participants were invited to the study site for their baseline visit and underwent a personal interview, standardized questionnaires and laboratory analysis. Germany also participated in the EuroAspire IV follow-up initiative that asked for a prospective follow-up about two years after the baseline visit [11]. This follow-up information was collected via a standardized telephone-based interview (see supplemental files).

### Definitions

Information on smoking status was missing for 59 participants in the medical record. To minimize drop-outs due to missing data, two variables from different time points were combined to define smoking status at index event: a) smoking status according to the medical record at index event, and b) current smoking one month prior to the index event as assessed retrospectively at the study baseline visit. Smoking status at baseline visit was confirmed by CO smokerlyser and the definition of smoking was “self-reported smoking and/or breath CO >10 ppm”. Smoking status at the time of the CHD index event (from medical record) and at the follow-up (by telephone interview) was bases on the “self-reported” smoking only. At the baseline visit, study participants reported their highest level of education; for this analysis, high school completed, college/university completed or post graduate degree were defined as high educational level. The cardiac event leading to the index hospitalization was used for classification (i.e., CABG, PCI, AMI, or AMIsch). Diabetes was defined according to ESC 2013/ADA 2012 criteria by plasma glucose ≥7.0 mmol/l or plasma glucose ≥11.1 mmol/l 2 h after standardized glucose load [12]. Depressed mood was assessed using the German version of the Hospital Anxiety and Depression Scale (HADS) questionnaire at the baseline visit. Its sub-test on depression uses seven items addressing depressive symptoms on an ordinal scale. Since all items have values ranging 0 to 3 the test for depression has a maximum score of 21. Presence of depressed mood was defined as sum score of eight or more [13]. At the baseline visit all participants were asked if they had been advised to attend a cardiac rehabilitation program within three months of discharge following the index event. Subsequently, the frequency of participation was assessed. Cardiac rehabilitation was counted as “participation”, if the participant had attended at least half of the recommended sessions. Data on the duration and type of the cardiac rehabilitation program (center-based vs home-based) were not systematically collected. In Germany, cardiac rehabilitation programs are usually center-based with a duration of three to four weeks.

### Outcome measure and data analysis

The primary endpoint of the current analysis was smoking status at the telephone-based follow-up interview amongst subjects classified as current smokers at the index event. Subsequently, the association between age, sex, educational level, coronary surgery at index event, diabetes, depressed mood and cardiac rehabilitation with smoking status at telephone-based follow-up interview was investigated. We compared smokers to non-smokers using statistical tests as described in the following. Continuous normally distributed variables are presented as mean (standard deviation) and analyzed by independent samples *t*-test. Nominal variables are presented as frequency (percentage) and analyzed by chi-squared test or Fisher’s exact test. A multivariable binary logistic regression model was built to illustrate effect sizes. Results are presented as odds ratios (OR) with 95% confidence intervals (95%CI) and *p*-values. Models were constructed block-wise: 1) sociodemographic variables (age, sex, educational level), 2) comorbidities (CABG at index event; diabetes or depressed mood at baseline visit), 3) participation in a cardiac rehabilitation program after the index event. Nagelkerke’s R^2^ values of respective models are presented to quantify goodness of fit. All analyses were performed using IBM SPSS Statistics, version 23.0 (IBM Corporation, NY, USA). Two-tailed *p*-values < 0.05 were considered statistically significant.

## Results

**Fig. 1:**
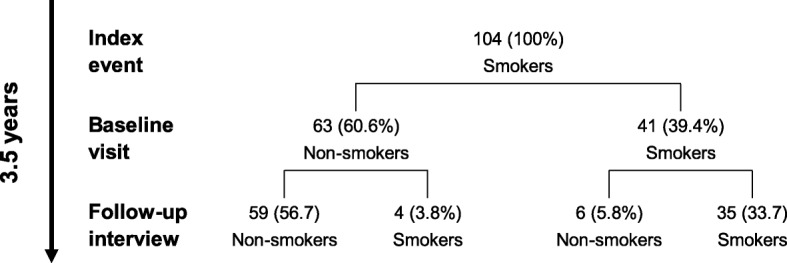
Frequency of smoking cessation in smokers with CHD. Index event occurred 6–36 months prior to baseline visit, and follow-up interview occurred about 2 years after baseline visit; median observation time between index event and telephone-based follow-up interview was 3.5 years (quartiles 3.0, 4.1)

Between August 2012 and March 2013, a total of 1380 persons were invited to participate in the German EuroAspire IV survey and 536 (38.8%) participated in the baseline visit. Baseline characteristics of the whole study population have been provided previously [14]. Of patients attending the baseline interview, 469 (87.5%) participated in the telephone-based follow-up interview. 124 of 536 (23.1%) patients were classified as current smokers at the index event. Of those, 104 (83.9%) participated in the telephone-based follow-up interview, thus, contributing to primary endpoint analyses (Fig. 1). The median time between index event and baseline visit was 1.8 years (quartiles 1.3, 2.4). Time between baseline visit and telephone-based follow-up interview was 1.8 years (quartiles 1.7, 1.8). Thus, the median total observation time for the 104 participants was 3.5 years (quartiles 3.0, 4.1). As compared to smokers at index event participating in the telephone-based follow-up interview (*n* = 104) non-participating smokers (*n* = 20) were older, more often diabetic and more often smokers at the baseline visit (all *p* < 0.05; see Supplemental table 1).

### Smoking cessation patterns

As presented in Fig. 1, 65 of 104 (62.5%) current smokers at index event had stopped smoking at telephone-based follow-up interview (median 3.5 years, see above). Of those, 63 (60.6%) participants quitted smoking between index event and baseline visit, but 4 (3.8%) subsequently relapsed until telephone-based follow-up interview. Late cessation was reported by 6 (5.8%) patients at telephone-based follow-up interview who were current smokers at index event and baseline visit. At the baseline visit 61 (64.2%) current smokers at index event reported to have received verbal advice to stop smoking following hospital discharge and 25 (26.3%) had been offered written information material. Recommendations to use pharmacological support (e.g. nicotine replacement therapy 16.8%, bupropion 0% or vareniclin 0%) were rarely reported. 26 (68.4%) current smokers at the baseline visit reported reduced smoking intensity since the index event but had not managed quitting. 19 of 39 (48.7%) persistent smokers at the telephone-based follow-up interview reported on any environmental tobacco smoke exposition (ETS) compared to 9 of 65 (13.8%) successful quitters. ETS was associated with reduced rates of smoking cessation (OR 0.16, 95%CI 0.06–0.42; *p* < 0.001; adjusted for age and sex). In the subgroup of smokers at index event not participating in a cardiac rehabilitation program but reporting ETS at baseline visit (*n* = 12), the rate of persistent smoking was 91.7%.

Seventy-six of 104 (73.1%) current smokers at index event reported having been advised to attend a cardiac rehabilitation program within three months of discharge (successful quitters vs persistent smokers at follow-up interview, 84.6% vs 53.8%, *p* = 0.001). Of those, 73 (96.1%) attended more than half of the recommended sessions (successful quitters vs persistent smokers at follow-up interview, 98.2% vs 90.5%, *p* = 0.12). Among participants of such a program, 67 (91.8%) received written educational materials, 69 (94.5%) took part in supervised exercise programs, 71 (97.3%) in health promotion workshops, and 62 (84.9%) in stress modification and relaxation interventions. Fourteen subjects (19.2%) participated in dedicated smoking cessation programs. Smokers reporting a quitting attempt prior to the index event were more likely to attend a cardiac rehabilitation program than smokers never reporting a quitting attempt (76.5% vs 58.3%; *p* = 0.05).

### Comparison of persistent smokers and successful quitters

The group of current smokers at index event was approximately 60 years old and predominantly male. Neither age, nor sex nor coronary surgery during index event showed a statistically significant association with subsequent smoking status, whereas a high educational level was associated with persistent smoking in univariable analysis (Table 1). Smoking status at index event was not associated with high educational level (smokers vs non-smokers at index event, 22.1% vs 21.1%, *p* = 0.82). Present diabetes showed a non-significant trend towards higher quitting rates, whereas absence of depressed mood and participation in a cardiac rehabilitation program were associated with smoking cessation in univariable analysis. A block-wise multivariable logistic regression model was built to search for factors determining smoking status at the telephone-based follow-up interview (Table 2). In the age-adjusted model (block one), higher educational level was associated with lower rates of smoking cessation. Extending the model to comorbidities (block two), presence of depressed mood was associated with lower rates of smoking cessation. Finally (block three), the strong influence of participation in a cardiac rehabilitation program was maintained the only significant factor associated with smoking cessation in patients with established CHD who were smoking at the time of index event (OR 5.19, 95%CI 1.87–14.46; *p* = 0.002). Goodness of fit (Nagelkerke’s R^2^) increased accordingly when adding explanatory variables although the explained variance remained small: 6.7, 16.6, 28.4% in blocks one, two and three, respectively. Additional variables potentially further elucidating the acuity of the index event (i.e., emergency admission and AMI) were not associated with smoking cessation (see Supplemental Table 2 and Supplemental Table 3).

**Table 1:** Current smokers at index event* (*n* = 104) stratified by their smoking status reported median 3.5 years later

	Total	Non-smokers at follow-up interview	Smokers at follow-up interview	
	***N*** = 104	***N*** = 65 (62.5%)	***N*** = 39 (37.5%)	***P***-value
**Demography**				
Age at index event, years	59.1 ± 9.0	59.5 ± 9.0	58.5 ± 9.1	0.61
Female sex	16 (15.4)	11 (16.9)	5 (12.8)	0.58
High educational level^#^	23 (22.1)	10 (15.4)	13 (33.3)	0.03
**Comorbidities**				
Type of index event: CABG	14 (13.5)	10 (15.4)	4 (10.3)	0.46
Diabetes (baseline)^a^	32 (31.1)	24 (37.5)	8 (20.5)	0.07
Depressed mood (baseline)^b^	25 (24.3)	11 (17.2)	14 (35.9)	0.03
**Intervention**				
Cardiac rehabilitation program after index event	73 (70.2)	54 (83.1)	19 (48.7)	< 0.001

Data are n (percent) or mean ± SD and p-values by asymptotic Pearson’s Chi-Squared test or independent sample t-test, as appropriate

*CABG* coronary artery bypass graft

*Index event occurred 6–36 months prior to baseline visit, and telephone-based follow-up interview occurred about 2 years after baseline visit; median observation time between index event and telephone-based follow-up interview was 3.5 years

^#^High school completed, college/university completed, postgraduate degree

^a^ Data missing for 1 participant

^b^ Data missing for 1 participant

**Table 2:** Factors associated with smoking cessation (block-wise multivariable logistic regression)

	Block 1		Block 2		Block 3	
Nagelkerke’s R squared	0.067		0.165		0.284	
	OR (95%CI)	P	OR (95%CI)	P	OR (95%CI)	P
**Demography**						
Age at index event*	1.02 (0.97–1.07)	0.40	1.01 (0.96–1.06)	0.66	1.03 (0.97–1.08)	0.36
Female sex	1.05 (0.32–3.44)	0.94	1.09 (0.31–3.89)	0.89	1.28 (0.34–4.77)	0.71
High educational level^#^	0.34 (0.13–0.91)	0.03	0.37 (0.13–1.03)	0.06	0.39 (0.13–1.17)	0.09
**Comorbidities**						
No CABG vs CABG			1.12 (0.28–4.52)	0.87	0.63 (0.15–2.68)	0.53
Diabetes (baseline)^a^			2.53 (0.93–6.86)	0.07	2.56 (0.89–7.34)	0.08
Depressed mood (baseline)^b^			0.32 (0.12–0.87)	0.03	0.37 (0.13–1.08)	0.07
**Intervention**						
Rehabilitation program					5.19 (1.87–14.46)	0.002

*OR* odds ratio, *95% CI* 95% confidence interval, *P P*-value, *CABG* coronary artery bypass graft

*OR per year

^#^High school completed, college/university completed, postgraduate degree

^a^ Data missing for 1 participant

^b^ Data missing for 1 participant

## Discussion

About one third of study participants were current smokers at the time of the index event. After a median observation period of 3.5 years, more than 60% successfully quitted smoking. Age, sex, and coronary surgery at index event were not associated with smoking cessation in this cohort. Lower educational level and absence of depression/depressed mood showed univariate associations with smoking cessation that were lost after multivariable adjustment. There, only attendance of a cardiac rehabilitation program was associated with successful smoking cessation.

Comparable studies such as the entire EuroAspire IV dataset [9], PREMIER [8], OASIS [4], and Olmsted County [15] reported smoking frequencies at an index event of 15–25%, and subsequent smoking cessation rates of about 50–65%, i.e. comparable to our findings. However, our analysis had the longest duration of follow-up. For the entire EuroAspire IV dataset one follow-up assessment of smoking status was done 6 to 36 months after the CHD index event (median 1.35 years; quartiles 0.95, 1.93); in PREMIER one assessment was done 6 month after AMI; the OASIS trial included one assessment 30 days and another 6 month after the diagnosis of unstable angina or AMI without ST-segment elevation; and Olmsted County reported quitting rates 6 month and 12 month after PCI. In our analysis smoking status was assessed at baseline visit 6 to 36 months after the CHD index event and again at telephone-based follow-up interview median 3.5 years (quartiles 3.0, 4.1) after the index event. Smoking rates at hospital admission and subsequent cessation rates after discharge apparently stagnated in Europe in the past decade, whereas smoking rates in patients aged below 50 years, especially in women, increased [6, 16]. A recent Cochrane review reported evidence of reduced admission rates for acute coronary syndrome and reduced mortality from smoking-related illnesses due to the implementation of World health organization recommended anti-smoking legislation initiatives [17, 18]. Results from Germany concur with these developments, but emphasize that reduced hospital admission rates for ST-elevation AMI in the period after implementing anti-smoking legislation were predominantly observed in non-smokers [19]. Reduced exposure to ETS by public smoking bans may reduce cardiovascular risk for non-smokers without measurably protecting smokers due to the prevailing effect of active smoking [19].

In our study, evidence-based treatment options for smoking cessation were rarely applied: 64.2% received verbal advice, 26.3% written information material and only 16.8% were advised to use nicotine replacement therapy, respectively. To be as effective as possible, smoking cessation schemes may include the comprehensive medical advice, behavioral aspects, community-oriented approaches and appropriate pharmacotherapeutic support [20, 21]. Any type of nicotine replacement therapy compared to control increases the chance of successful quitting by 50–70% according to a systematic Cochrane review [22]. Nevertheless, throughout Europe, smoking cessation counselling and medication remains heavily underused in clinical practice [9, 23]. Half of German general practitioners reported low activity in smoking cessation promotion [24]. Hence, the barriers to smoking cessation need to be addressed comprehensively. Exposure to ETS was more common in persistent smokers than successful quitters in this cohort, especially at home. Continuous exposure to tobacco smoke may impede successful cessation and foster relapse.

Higher levels and longer periods of education were shown to be associated with non-smoking, improvements in cardiovascular risk factor control, smoking cessation in CHD patients and smoking cessation in healthy youth and adults smokers [25–28]. Surprisingly, in this cohort of 104 pre-CHD-event smokers, there was a univariate association of higher educational level with persistent smoking, that lost significance in the multivariable model. By contrast, smoking status at index event was not associated with higher educational level. Higher educational level was defined by high school completed, college/university completed or postgraduate degree. Supporting this finding, young Chinese male adult smokers with higher levels of education, defined by ≥ senior college or university, were more inclined to smoke, despite a better knowledge on tobacco related health hazards [29]. The definition of higher education is subject to variation: more than primary school [26], more than high school [27] or three ordinal categories such as primary, secondary and tertiary education [25, 28] are used to define educational level in this context. As 103 of 104 German pre-event smokers had more than primary school leaving qualification, the observed association vanished when higher education was defined by more than primary school. Nevertheless, increased rates of persistent smoking in the subgroup of pre-event smokers with very high school leaving qualification are a reason for concern.

In our study, there was an association of depressed mood with lower quitting rates in patients with CHD in univariable analysis that was lost after multivariable adjustment. Comparable studies with higher sample size had reported a significant association [8]. Both smoking and depression/depressed mood were shown to negatively impact on cardiovascular mortality, morbidity and quality of life [30]. The relationship between depression/depressed mood and smoking in CHD includes a bidirectional influence, as well as genetic determination [31]. Although it is yet unknown whether routine screening for psychosocial risk factors may reduce future cardiac events, the negative influence of depression/depressed mood on both smoking cessation and CHD is commonly acknowledged. According to current guidelines physicians were encouraged to screen smokers with CHD for comorbid depression/depressed mood. It is plausible that successful targeting depression may raise chances of successful smoking cessation [5].

The rates of participation in a cardiac rehabilitation program in the German part of EuroAspire IV were higher than average, especially for smokers at index event [9, 32, 33]. Attending a cardiac rehabilitation program was shown to improve successful cessation and lower mortality rates in systematic reviews and meta-analysis of randomized controlled trials [5, 34–37]. According to a recent analysis based on a Cochrane review, 58% of the beneficial effects of cardiac rehabilitation programs were attributable to changes in cardiovascular risk factors (i.e., smoking, lipids, blood pressure) and 24% of the total mortality risk reduction might be accountable to reduced smoking [38]. However, the analysis of the entire EuroAspire IV data set showed no association of smoking cessation with survival in the telephone-based follow-up interview period [11]. Though pre-event smokers were less likely to participate, the entire EuroAspire IV data set showed a positive correlation of participating in a cardiac rehabilitation program and quitting smoking at baseline visit 6 to 36 months after the CHD index event [26, 39]. Supporting this findings, we observed a strong correlation of participating in a cardiac rehabilitation program and quitting at telephone-based follow-up interview median 3.5 years after the CHD index event. The effect size found in the present study relating participation in a cardiac rehabilitation program and successful smoking cessation appears high compared to published literature [8, 15]. Nevertheless, these findings support the importance of cardiac rehabilitation programs in secondary prevention of CHD, thus encouraging physicians to more actively advocate this strategy.

### Limitations and strengths

The EuroAspire surveys rely on defined geographical areas [9]. Hence, patient characteristics and treatment quality may have differed from CHD populations in other regions in Germany. Furthermore, participation rate in the baseline interview was low, which is in accordance with the overall trend of declining response rates during the past decades [40]. The sample size was too small to verify or disprove the non-significant associations of education and depressed mood with smoking cessation or enrich the multivariable model with additional variables such as retrospective characteristics of smoking history, comorbidities or cessation efforts. The overall explanatory power of the multivariable model was low due the small sample size. This reflects the complexity of predicting behavioral change adequately. Smoking status was assessed by self-report by accepted methods (reported sensitivity 87.5%, specificity 89.2% [41];). The duration and intensity of smoking (i.e., pack years) was not documented in the medical records at index event obtained retrospectively and could not reliably be recorded during the interview due to recall bias.

Strengths of the current analysis include the use of standardized comparable methods ranging from medical record data extraction forms, questionnaires and centralized laboratory assessments. The personal interviews provided detailed and complete risk factor recording, which comparable studies based on medical records alone often lacked. A small but very high-risk sample was studied in matters of the modifiable risk factor tobacco smoking.

## Conclusions

Smoking rates at a cardiac event, as well as, persistent smoking rates were high in this cohort. Evidence-based options supporting smoking cessation such as comprehensive medical counseling and nicotine replacement therapy were underused. Referral to a cardiac rehabilitation program was strongly associated with smoking cessation. Referral rates were higher than average in the subgroup of smokers, but coverage remained incomplete. Cardiologists and general practitioners in hospitals and primary care facilities should be motivated to enhance non-pharmacological risk factor control, especially smoking cessation counselling and referral to a cardiac rehabilitation program as important means to improve the success of secondary prevention efforts.

## Supplementary information

**Additional file 1: Supplemental Table 1.** Current smokers at index event* (*n* = 124) stratified by their participation at telephone-based follow-up interview. **Supplemental Table 2.** Clinical data of current smokers at index event* (*n* = 104) stratified by their smoking status reported 3.5 years later. **Supplemental Table 3.** Factors associated with smoking cessation (block-wise multivariable logistic regression).

**Additional file 2.** EuroAspire IV Germany I Follow-up interview I Questionnaire.

## Data Availability

All data that support the findings of this study are included in this published article. The datasets used and/or analysed during the current study are available from the corresponding author on reasonable request.

## Abbreviations

CHD: Coronary heart disease
EuroAspire IV: European action on secondary and primary prevention by intervention to reduce events
CABG: Coronary artery bypass graft
PCI: Percutaneous coronary intervention
AMI: Acute myocardial infarction
AMIsch: Acute myocardial ischemia without infarction
HADS: Hospital anxiety and depression scale
ETS: Environmental tobacco smoke
