# Supplementary material for: Factors associated with smoking cessation in patients with coronary heart disease: a cohort analysis of the German subset of EuroAspire IV survey
Source: BMC Cardiovasc Disord. 2020 Mar 30;20:152. doi: 10.1186/s12872-020-01429-w (PMC7106891; doi:10.1186/s12872-020-01429-w)
Supplement: Supplementary file 1 — Additional file 1: Supplemental Table 1. Current smokers at index event* (n = 124) stratified by their participation at telephone-based follow-up interview. Supplemental Table 2. Clinical data of current smokers at index event* (n = 104) stratified by their smoking status reported 3.5 years later. Supplemental Table 3. Factors associated with smoking cessation (block-wise multivariable logistic regression). [file 12872_2020_1429_MOESM1_ESM.docx]

# Supplementary material

Supplemental Table 1 Current smokers at index event* (n=124) stratified by their participation at telephone-based follow-up interview

|  | **Total** | **Participants at follow-up interview** | **Non-participants at follow-up interview** |  |
| --- | --- | --- | --- | --- |
|  | **N=124** | **N=104 (83.9%)** | **N=20 (16.1%)** | **P-value** |
| **Demography** |  |  |  |  |
| Age at index admission, years | 59.8±9.1 | 59.1±9.0 | 63.6±9.3 | <0.05 |
| Female sex | 19 (15.3) | 16 (15.4) | 3 (15.0) | 0.97 |
| High educational level^#^ | 26 (21.0) | 23 (22.1) | 3 (15.0) | 0.47 |
| **Comorbidities** |  |  |  |  |
| Current smoker at baseline visit | 54 (43.5) | 41 (39.4) | 13 (65.0) | 0.04 |
| Type of index event: CABG | 18 (14.5) | 14 (13.5) | 4 (20.0) | 0.45 |
| Diabetes (baseline)^a^ | 44 (36.1) | 32 (31.1) | 12 (63.2) | <0.01 |
| Depressed mood (baseline)^b^ | 32 (26.0) | 25 (24.3) | 7 (35.0) | 0.32 |
| **Intervention** |  |  |  |  |
| Cardiac rehabilitation program after index event | 86 (69.4) | 73 (70.2) | 13 (65.0) | 0.65 |
| Data are n (percent) or mean±SD and p-values by asymptotic Pearson’s Chi-Squared test or independent sample t-test, as appropriate.  CABG, coronary artery bypass graft.  *Index event occurred 6-36 months prior to baseline visit, and telephone-based follow-up interview occurred about 2 years after baseline visit; median observation time between index event and telephone-based follow-up interview was 3.5 years.  ^#^High school completed, college/university completed, postgraduate degree.  ^a^ Data missing for 1 participant.  ^b^ Data missing for 1 participant. | | | | |

Supplemental Table 2 Clinical data of current smokers at index event* (n=104) stratified by their smoking status reported 3.5 years later

|  |  | **Total** | **Non-smokers**  **at follow-up interview** | **Smokers**  **at follow-up interview** |  | |
| --- | --- | --- | --- | --- | --- | --- |
|  |  | **N=104** | **N=65 (62.5%)** | **N=39 (37.5%)** | **P-value** | |
| Type of index event: CABG | | 14 (13.5) | 10 (15.4) | 4 (10.3) | 0.46 | |
| Type of index event: emergency^a^ | | 65 (63.1) | 41 (64.1) | 24 (61.5) | 0.80 | |
| Type of index event: infarction | | 61 (58.7) | 41 (63.1) | 20 (51.3) | 0.24 | |
| Data are n (percent) and p-values by asymptotic Pearson’s Chi-Squared test.  CABG, coronary artery bypass graft.  *Index event occurred 6-36 months prior to baseline visit, and telephone-based follow-up interview occurred about 2 years after baseline visit; median observation time between index event and telephone-based follow-up interview was 3.5 years.  ^a^ Data missing for 1 participant. | | | | | |  |

Supplemental Table 3 Factors associated with smoking cessation (block-wise multivariable logistic regression)

|  | **Block 1** | | **Block 2** | | **Block 3** | |
| --- | --- | --- | --- | --- | --- | --- |
|  | OR (95%CI) | P | OR (95%CI) | P | OR (95%CI) | P |
| **Demography** | | | | | | |
| Age at index event* | 1.02 (0.97-1.07) | 0.43 | 1.02 (0.97-1.07) | 0.38 | 1.02 (0.98-1.07) | 0.33 |
| Female sex | 1.06 (0.32-3.49) | 0.93 | 1.03 (0.31-3.42) | 0.96 | 1.22 (0.35-4.25) | 0.75 |
| High educational level^#^ | 0.33 (0.12-0.89) | 0.03 | 0.32 (0.12-0.88) | 0.03 | 0.34 (0.12-0.92) | 0.03 |
| **Clinical data** | | | | | | |
| No CABG vs CABG | 1.72 (0.48-6.21) | 0.41 | 1.70 (0.45-6.41) | 0.43 | 1.69 (0.45-6.37) | 0.44 |
| Elective vs emergency^a^ |  |  | 1.35 (0.56-3.24) | 0.50 | 1.01 (0.37-2.75) | 0.98 |
| Ischemia vs infarction |  |  |  |  | 1.80 (0.68-4.80) | 0.24 |
| OR, odds ratio; 95%CI, 95% confidence interval; P, P-value; CABG, coronary artery bypass graft.  *OR per year.  ^#^High school completed, college/university completed, postgraduate degree.  ^a^ Data are missing for 1 participant. | | | | | | |
