# Supplementary material for: Factors associated with smoking cessation in patients with coronary heart disease: a cohort analysis of the German subset of EuroAspire IV survey
Source: BMC Cardiovasc Disord. 2020 Mar 30;20:152. doi: 10.1186/s12872-020-01429-w (PMC7106891; doi:10.1186/s12872-020-01429-w)
Supplement: Supplementary file 2 — Additional file 2. EuroAspire IV Germany I Follow-up interview I Questionnaire. [file 12872_2020_1429_MOESM2_ESM.pdf]

Participant-ID:

EuroAspire IV Germany
Follow-up interview
German original version transcribed to English (March 2020)

Vital status				
**V1. Date of follow-up interview**	____/____/____ DD/MM/YYYY			
**V2. Vital status**	☐ dead	☐ alive__________________________	☐ uncertain	☐ no response

If the participant has died

**V3. Date of death**	____/____/____ DD/MM/YYYY			
**V4. Source of information**	☐ relatives	☐ registration office	☐ treating institution	☐ other source, see free text __________________________
**V5. Cause of death**	☐ cardiovascular<br>☐ malignant disease<br>☐ uncertain, see free text<br>__________________________	☐ cerebrovascular<br>☐ infection/sepsis<br>☐ no response	☐ other vascular<br>☐ other cause, see free text<br>__________________________	

Participant-ID:

Re-hospitalization and comorbidities

**Please identify the date of the baseline interview in the data bank and include this date in the following questions.**		**If yes, date of admission to hospital:**	**If yes, has it been an emergency?**
**K1. Since the date of the baseline interview, have you been admitted to a hospital due to a heart attack?**	☐ yes<br>☐ no<br>☐ uncertain<br>☐ no response	____/_____<br>MM/ YYYY<br>*or*<br>☐ first half of the year<br>☐ second half of the year<br>☐ uncertain<br>☐ no response	☐ yes<br>☐ no<br>☐ uncertain<br>☐ no response
**K2. Since the date of the baseline interview, have you been admitted to a hospital due to a percutaneous coronary intervention?**	☐ yes<br>☐ no<br>☐ uncertain<br>☐ no response		☐ yes<br>☐ no<br>☐ uncertain<br>☐ no response
**K3. Since the date of the baseline interview, have you been admitted to a hospital due to a coronary artery bypass graft operation?**	☐ yes<br>☐ no<br>☐ uncertain<br>☐ no response		☐ yes<br>☐ no<br>☐ uncertain<br>☐ no response

**Since the date of the baseline interview, has your doctor told you, that … :**		**When have you been told?**	**Since the date of the baseline interview, have you been admitted to a hospital due to that diagnosis?**	**If yes, date of admission to hospital:**	**If yes, has it been an emergency?**
**K4. you have diabetes (diabetes mellitus)?**	☐ yes<br>☐ yes, medical record<br>☐ no<br>☐ uncertain<br>☐ no response	____/_____<br>MM/ YYYY	☐ yes<br>☐ no<br>☐ uncertain<br>☐ no response	____/_____<br>MM/ YYYY	☐ yes<br>☐ no<br>☐ uncertain<br>☐ no response

Participant-ID:

**K5. you have chronic kidney disease (impairment of renal function)?**	☐ yes<br>☐ yes, medical record<br>☐ no<br>☐ uncertain<br>☐ no response	___/____<br>MM/ YYYY	☐ yes<br>☐ no<br>☐ uncertain<br>☐ no response	___/____<br>MM/ YYYY	☐ yes<br>☐ no<br>☐ uncertain<br>☐ no response
If K5 yes,	K5a. Are you dependent upon dialysis treatment?		☐ yes<br>☐ no<br>☐ uncertain<br>☐ no response	If K5a yes, since when have you been dependent upon dialysis treatment<br>___/_____MM/ YYYY	
	K5b. Did you have a kidney transplant?		☐ yes<br>☐ no<br>☐ uncertain<br>☐ no response	If K5b yes, when did you have a kidney transplant<br>___/_____MM/ YYYY	
**K6. you have COPD (chronic obstructive pulmonary disease)?**	☐ yes<br>☐ yes, medical record<br>☐ no<br>☐ uncertain<br>☐ no response	___/____<br>MM/ YYYY	☐ yes<br>☐ no<br>☐ uncertain<br>☐ no response	___/____<br>MM/ YYYY	☐ yes<br>☐ no<br>☐ uncertain<br>☐ no response
**K7. you have PAD (peripheral arterial occlusive disease)?**	☐ yes<br>☐ yes, medical record<br>☐ no<br>☐ uncertain<br>☐ no response	___/____<br>MM/ YYYY	☐ yes<br>☐ no<br>☐ uncertain<br>☐ no response	___/____<br>MM/ YYYY	☐ yes<br>☐ no<br>☐ uncertain<br>☐ no response

Participant-ID:

Since the date of the baseline interview, has your doctor told you, that … :		When have you been told?	Since the date of the baseline interview, have you been admitted to a hospital due to that diagnosis?	If yes, date of admission to hospital:	If yes, has it been an emergency?
**K8. you have a stroke or TIA (transient ischemic attack)?**	☐ yes<br>☐ yes, medical record<br>☐ no<br>☐ uncertain<br>☐ no response	___/____<br>MM/ YYYY	☐ yes<br>☐ no<br>☐ uncertain<br>☐ no response	___/____<br>MM/ YYYY	☐ yes<br>☐ no<br>☐ uncertain<br>☐ no response
If K8 yes,	K8a. What was the cause of stroke?	☐ ischemia ☐ hemorrhage ☐ uncertain ☐ no response			
**K9. you have heart failure?**	☐ yes<br>☐ yes, medical record<br>☐ no<br>☐ uncertain<br>☐ no response	___/____<br>MM/ YYYY	☐ yes<br>☐ no<br>☐ uncertain<br>☐ no response	___/____<br>MM/ YYYY	☐ yes<br>☐ no<br>☐ uncertain<br>☐ no response
**K10. you have a tumor?**	☐ yes<br>☐ yes, medical record<br>☐ no<br>☐ uncertain<br>☐ no response	___/____<br>MM/ YYYY	☐ yes<br>☐ no<br>☐ uncertain<br>☐ no response	___/____<br>MM/ YYYY	☐ yes<br>☐ no<br>☐ uncertain<br>☐ no response
If K10 yes,	K10a. Where is your tumor localized? Is your tumor malignant or benign?	☐ see free text<br>______________			

Participant-ID:

Smoking patterns

**S1. Have you ever been smoking regularly?**<br>If yes, continue with S2<br>If no, go to **non-smoker** page 9	☐ yes<br>☐ no<br>☐ uncertain<br>☐ no response
**S2. Do you smoke currently?**<br>If yes, got to **current smoker** page 6<br>If no, got to **former smoker** page 7	☐ yes<br>☐ no<br>☐ uncertain<br>☐ no response

Julius-Maximilians-
UNIVERSITÄT
WÜRZBURG

Participant-ID:

Questions to current smokers	
**S3. Currently, how many cigarettes do smoke regularly at one day?**	_________ cigarettes / day<br>☐ uncertain<br>☐ no response
**S4. Currently, what is the time between getting up and first cigarette in the morning?**	☐ x <= 5 min<br>☐ 5min < x <= 30min<br>☐ 30 min < x <= 60 min<br>☐ x => 60 min<br>☐ uncertain<br>☐ no response
**S5. Have you ever been non-smoking for a limited period?**<br>If yes, go to **additional questions** page 8<br>If no, read out the following to the study participant and continue with SW1	☐ yes<br>☐ no<br>☐ uncertain<br>☐ no response
"In the present survey we are interested in understanding your personal motives with handling tobacco smoking."	
**SW1. Would you please try to describe your most important reasons to smoke?**<br>**(Wait for answer and continue with the following)**<br>Are there reasons you consider positive, such as pleasure, joy or relaxation or even reasons you consider negative, such as addiction or fear of withdrawal symptoms?<br>**END**	☐ see free text<br>___________________________________<br>___________________________________<br>___________________________________<br>☐ uncertain<br>☐ no response

Participant-ID:

Questions to former smokers

**S3. How many cigarettes have you been smoking regularly one week before quitting smoking?**	_________ cigarettes / day<br>☐ uncertain<br>☐ no response
**S4. What was the time between getting up and first cigarette in the morning one week before quitting smoking?**	☐ x <= 5 min<br>☐ 5min < x <= 30min<br>☐ 30 min < x <= 60 min<br>☐ x => 60 min<br>☐ uncertain<br>☐ no response
**S6. When did you quit smoking?**	_________ age<br>_________ year<br>☐ uncertain<br>☐ no response
**S7. Did you quit smoking from one day to the next?**	☐ yes<br>☐ no<br>☐ uncertain<br>☐ no response
**S8. Have you ever had been non-smoking for a limited period, before you quitted smoking?**<br>If yes, go to **additional questions** page 8<br>If no, read out the following to the study participant and continue with SW4	☐ yes<br>☐ no<br>☐ uncertain<br>☐ no response
"In the present survey we are interested in understanding your personal motives with handling tobacco smoking."	
**SW4. Would you please try to describe the reasons why you stopped smoking definitely?**<br>**(Wait for answer and continue with the following)**<br>Did health risks for you or others, financial issues or important life events influence your decision?	☐ see free text<br>___________________________________<br>___________________________________<br>___________________________________<br>☐ uncertain<br>☐ no response
If no diagnosis / disease was mentioned in SW4, **END**<br>If a diagnosis / disease was mentioned in SW4, go to SW4b	
**SW4b. Did you consider this diagnosis / disease life-threatening?**<br>**END**	☐ yes<br>☐ no<br>☐ uncertain<br>☐ no response

Participant-ID:

Additional questions for limited periods of non-smoking

Read out the following to the study participant:

"If there is more than one period of non-smoking, please remember your longest period of non-smoking"

**S9. How long has that period been?**	☐ x <= 3 months<br>☐ 3 < x <= 6 months<br>☐ 6 < x <= 12 months<br>☐ x > 12 months<br>☐ free text<br>☐ uncertain<br>☐ no response
**S10. When did that period begin?**	At ________ years of age<br>In the year ______<br>☐ uncertain<br>☐ no response
**S11. Did you stop smoking at that time from one day to the next?**	☐ yes<br>☐ no<br>☐ uncertain<br>☐ no response

Read out the following to the study participant:

"In the present survey we are interested in understanding your personal motives with handling tobacco smoking."

**SW2. Would you please try to describe the reasons why you tried to stop smoking at that time?**<br>**(Wait for answer and continue with the following)**<br>Did health risks for you or others, financial issues or important life events influence your decision?	☐ see free text<br>______________________________<br>______________________________<br>☐ uncertain<br>☐ no response

If no diagnosis/disease was mentioned in SW2, go to SW3

If a diagnosis/disease was mentioned in SW2, go to SW2b

**SW2b. Did you consider this diagnosis / disease life-threatening?**	☐ yes<br>☐ no<br>☐ uncertain<br>☐ no response
**SW3. Would you please try to describe the reasons, why you restarted smoking at that time?**<br>**(Wait for answer and continue with the following)**<br>Has there been an extraordinary situation or event?	☐ see free text<br>______________________________<br>______________________________<br>☐ uncertain<br>☐ no response

If the study participant is a **current smoker**, go to SW1 page 6

If the study participant is a **former smoker**, go to SW4 page 7

Participant-ID:

Questions to non-smokers

Read out the following to the study participant:<br>"In the present survey we are interested in understanding your personal motives with handling tobacco smoking."	
**SW5. Would you please try to describe the reasons why you never started smoking?**<br>**(Wait for answer and continue with the following)**<br>Did health risks for you or others, financial issues or important life events influence your decision?<br>**END**	☐ see free text<br>________________________________<br>________________________________<br>☐ uncertain<br>☐ no response
